# Prevalence and Gender-Specific Influencing Factors of Hypertension among Chinese Adults: A Cross-Sectional Survey Study in Nanchang, China

**DOI:** 10.3390/ijerph15020382

**Published:** 2018-02-23

**Authors:** Hui Zhou, Kai Wang, Xiaojun Zhou, Shiying Ruan, Shaohui Gan, Siyuan Cheng, Yuanan Lu

**Affiliations:** 1 Jiangxi Province Key Laboratory of Preventive Medicine, School of Public Health, Nanchang University, Nanchang, Jiangxi 330006, China; zhouhui115658@163.com (H.Z.); rsy@nuc.edu.cn (S.R.); g695297@163.com (S.G.); 2 School of Medicine, Nanchang University, Nanchang, Jiangxi 330006, China; 416523216406@email.ncu.edu.cn; 3 Department of Public Health Sciences, University of Hawaii at Mānoa, Honolulu, HI 96822, USA; 313279@stu.wvusd.org

**Keywords:** hypertension, influencing factors, prevalence, gender-specific, China

## Abstract

Hypertension has become the leading cause of death worldwide; data on hypertension among Nanchang adults are sparse. The aim of this study was to investigate the prevalence and gender-specific influencing factors of hypertension in adults in Nanchang, China. A cross-sectional survey was conducted with a representative sample of 2722 Chinese residents aged 18 years and above between May and September 2016, with a response rate of 92.4% (2516/2722). A stratified cluster sampling method was adopted in this study. Data on prevalence and influencing factors were obtained from a standard questionnaire and physical measurements. Univariate and multivariate logistic regressions were performed to analyze the influencing factors. The age-standardized prevalence was 19.8% (18.2–21.3) (male: 19.5% (18.0–21.1); female, 20.01% (18.5–21.6)). Factors positively associated with hypertension prevalence were past smoking, diabetes mellitus (DM), and overweight and obesity in both genders. Abdominal obesity and family history of cardiovascular diseases (CVD) were risk factors only in males; sleeping time and consumption of fresh vegetables and fruits were related to the prevalence of hypertension only in females. These findings will form the baseline information for the development of more effective approaches to enhance current prevention and control management of hypertension.

## 1. Introduction

According to the world health statistics report of 2015, hypertension prevalence among adults aged 18 years and older was 22.2% in 2014; Africa was identified to be the region with the highest prevalence (29.6%) [1]. In China, hypertension has been a more serious chronic disease, which affected approximately 160 million (18.8%) to 337 million Chinese (33.5%) during 2002–2010 [2,3]. According to the latest Chinese report on cardiovascular disease in 2015, there were 270 million people (25.2%) living with hypertension in 2012, indicating that at least 2 in 10 adults had hypertension; the total death due to hypertension was over 2 million (24.6%) in China [4]. In comparison, the global deaths attributed to high blood pressure in adults were 12.8% each year [5]. In 2013, the direct economic burden caused by hypertension made up 6.6% of the total health costs in China [4]. Hypertension has become an important public health concern, due to high prevalence, strong associations with cardiovascular diseases (CVD), relation with morbidity and mortality, and huge economic burdens on the family and society [6,7].

Nanchang is provincial capital of Jiangxi province located in the southeast of China. Jiangxi province is one of the poorest provinces in China with a gross domestic product (GDP) per capita of only ¥36,724 (about US$5923) in 2015 [8]. Health conditions and facilities are relatively poor, and little research has been conducted to study the prevalence and influencing factors of hypertension among adults in Nanchang. Although it has been widely reported that there is a significant discrepancy between the prevalence rate of hypertension among different genders, efforts in analyzing the gender differences of influencing factors associated with hypertension have been very limited [6,9].

Hence, the main aims of the present study were to estimate the prevalence to identify the gender-specific influencing factors of hypertension and to evaluate these associations by performing household surveys among adult residents in Nanchang city.

## 2. Materials and Methods

### 2.1. Context of the Study Setting

Nanchang is the capital of the Jiangxi province, located in the southeast inland of China and south of the Yangtze River, with a population of 5.37 million and a total area of 7402.4 km^2^; Nanchang includes six municipal districts, three developing districts, and three counties [10]. Qingyunpu, a District of Nanchang with 320,000 inhabitants (male: 51.6%; female: 48.4%), was selected as representative reference for this study because of its socio-economic status in relation to that of overall city of Nanchang [11].

### 2.2. Study Design and Sample Size

A cross sectional survey was conducted with residents aged 18 years and above in Qingyunpu District during May and September 2016. In this study, the sample size was calculated by the formula

(1) $$n=deff\frac{Z_{\alpha }^{2}P\left(1-P\right)}{\delta^{2}}$$

where n is the sample size; deff is the sampling design efficiency, which was set it at 2.0 in this study; $Z_{\alpha }^{2}$ is the abscissa of the normal curve that cuts off an area at the tails (1-α equals the desired confidence level, e.g., 95%); P is based on the prevalence of 24.4%, which was the prevalence of chronic diseases in Nanchang city, in 2013; and δ is the desired level of precision, assigned at 10% [12,13]. Thus, the sample size was calculated as

(2) $$n=2.0\times \frac{2^{2}\times 0.244\times (1-0.244)}{{\left(0.244\times 10\%\right)}^{2}}\approx 2479$$

Considering that there may be potential non-response rate, which we desired to be less than 10%, therefore we distributed a total of 2722 questionnaires. There should be adequate power at all waves.

### 2.3. Sampling Methods and Recruiting Standards

A multi-staged and stratified cluster sampling method was utilized in this study. In the first stage, three sub-districts were randomly selected from the total of seven sub-districts within Qingyunpu District; in the second stage, simple random sampling was used to select one community or village from each sub-district, and two communities and one village were selected for each sub-district; thirdly, cluster sampling was used for random extraction of participants from the selected communities and villages in this study. A total of 2722 adults who have lived in the district for more than six months were included. Exclusion criteria included subjects suffering from some severe diseases, having fever in the last 15 days, or who were currently pregnant; the final stage of this survey study included two phases: Phase 1 recruited the participants to complete a questionnaire administered by door-to-door interviews; and Phase 2 required participants to complete physical measurements.

### 2.4. Date Collection and Survey Variables

A structured questionnaire was designed based on the “Basic Schema and Data Standard of Health Record (trial)” [14]. The standardized questionnaire was administered via door-to-door interviews by trained undergraduate and postgraduate students from the school of public health at Nanchang University. These trained students also measured the height, weight, and waist circumference for each participant in households according to the standard procedure [15,16]. Participant data were collected and stored using a personal computer within 24 h following the interview.

The questionnaire acquired related information of various activities from the participants including: walking, cycling, going up and down, climbing, ballroom dancing, morning exercises, aerobics, gymnastics, tai chi, yoga, volleyball, badminton, table tennis, golf, tennis, basketball, running, jumping rope, swimming, bowling, and other activities of the average daily accumulated activity time. The 1000-step equivalent time refers to the amount of time it takes for an activity to complete an amount equal to one thousand steps [17]. According to “the National Manual of Assessment and Evaluation of the Comprehensive Prevention and Control of Chronic Non-Communicable Diseases", we can see the average number of daily activities of residents thousands of steps = average activity time/one thousand steps equivalent time; National Chronic Non-Communicable Diseases Prevention and Control Working Group based on ≥6000 stride/d for the standards of fitness [18].

The dependent variable in this study was hypertension. We considered hypertension as any participant with systolic blood pressure ≥140 mmHg and/or diastolic pressure ≥90 mmHg, a self-reported history of hypertension from themselves, or taking any anti-hypertension drugs in the past two weeks [19].

The independent variables included: demographics (age, location, marital status, educational level, and occupation), behavioral measures (smoking, alcohol drinking, physical exercise, sleeping time and daily consumption of salt, vegetables and fruits), physical measures (DM (diabetes mellitus), body mass index, abdominal obesity), and family history of CVD. Some independent variables were transformed, recoded, and grouped for analytical purposes. Table 1 summarizes the different transformations that were carried out.

### 2.5. Statistical Analysis

EpiData (version 3.1, CDC, Atlanta, GA, USA) was used to collect data and create the database. Statistic Package for Social Science software release 18.0 (SPSS Inc., Chicago, IL, USA) and Microsoft Excel 2010 (Microsoft Ltd., Washington, DC, USA) were used for statistical analyses. Analyses were carried out separately for male and female adults. The categorical variables were described as numbers of cases and proportions. Continuous variables were first tested for normality. If the variables obey the normal distribution, they were presented as mean with standard deviation, otherwise they were presented as median values with quartiles. The direct standardized methods were used to calculate age-standardized prevalence of hypertension for all participants in different age groups based on the 2010 China population census data [22]. We computed 95% CI of prevalence rates as

(3) $$95\%\mathrm{CI}\;=P\pm Z_{\propto }\times \;\surd \left[P\;\left(1-P\right)/N\right]$$

where P and N are the rate and the number of individuals; $Z_{\propto }$ is the abscissa of the normal curve that cuts off an area at the tails (1-α equals the desired confidence level, e.g., 95%), we assigned it as 1.96.

The Chi-square test was used for comparisons of categorical variables among groups. The Student’s t-test was performed to analyze continuous variables with a normal distribution and the rank sum test was performed to analyze continuous variables without normal distribution. In this study, all continuous variables did not obey normal distribution, so only the rank sum test was used. Odds ratios (ORs) with 95% confidence intervals (CIs) were calculated by univariate and multivariate logistic regression to evaluate the influencing factors of hypertension. Two-sided *p* < 0.05 was considered to be statistically significant.

### 2.6. Ethical Statement

The procedures employed were in accordance with the ethical standards of the Helsinki Declaration (1975, amended most recently in 2008) of the World Medical Association. Participants provided informed consent prior to data collection and their personal information was maintained anonymously. This study was approved by the Jiangxi Humanities and Social Sciences Institutional Review board (GL162033).

## 3. Results

### 3.1. Characteristics of the Study Population

A total of 2722 subjects participated in the study, and 2516 participants who completed the questionnaire and measurements were enrolled for data analysis showing a response rate of 92.4%. Table 2 shows the characteristics of participants stratified by gender. There were no statistically significant differences between males and females in location, family history of CVD, and salt consumption. All of the remaining variables were significantly higher in females than in males, except for sleeping time, smoking history, and alcohol consumption, which were higher in males.

### 3.2. Prevalence and Age-Standardized Hypertension Prevalence

Given a crude prevalence rate of 28.7% (722/2516) (males: 27.2% (337/1238); females: 30.1% (385/1278)), the age-standardized prevalence was 19.8% (males: 19.5%; females: 20%). Even though the hypertension was little more prevalent in females than in males, this difference was not statistically significant (*p* > 0.05). As shown in Table 3, the hypertension prevalence for all the participants appeared to increase significantly with age.

### 3.3. Univariate Analysis for Influencing Factors of Hypertension in Different Gender

Table 4 displays the number of participants and its proportions, hypertensive and its prevalence, crude odds ratio (OR) and its CI for males and females. In the male model, it shows that many factors were associated with increased odds for hypertension except for alcohol consumption and daily salt intake. In the female model, most variables were in the same direction as the males except that physical exercise was a statistically insignificant factor.

### 3.4. Multivariate Analysis for Influencing Factors of Hypertension in Males and Females

Our study examined gender-specific influencing factors of hypertension. Two kinds of logistic regression models were developed to analyze the influencing factors. Model 1 was conducted by examining many different influencing factors possibly related to hypertension and the model 2 was performed by inclusion of location, marital status, educational level, and occupation to the analysis. Both these analyses showed no statistically significant interaction between these factors and hypertension.

As summarized in Table 5, the multivariate logistic regression model 2 revealed that aging, smoking history, DM, and high BMI were related with higher odds for having hypertension in both genders. While these influencing factors were shown to increase the odds for hypertension in both genders, the magnitude of this effect differed. Females with previous smoking history had an increased likelihood of getting hypertension compared to males ((OR: 11.425; CI: 1.873–69.682) vs. (OR: 1.921; CI: 1.090–3.384)). Females who were diabetic were less likely to suffer from hypertension than males ((OR: 20.202, CI: 10.896–37.456) vs. (OR: 41.192; CI: 15.720–107.936)). However, abdominal obesity and family history of CVD were positively associated with prevalence only in males, while amount of sleep per night and daily consumption of vegetables and fruits were related to the prevalence only in females.

## 4. Discussion

This study discovered that the age-standardized prevalence of hypertension in Jiangxi (19.8%) was lower than the 22.2% from the latest report from world health statistics among adults (2015) [1]. The rate was also lower than those reported from the United States, some European countries, and South and East Asian countries besides Japan [24,25,26,27,28,29,30]. Compared to some studies done in China, the hypertension rate from this study was much lower than the reported rate of 29.6% (male: 31.2%; female: 28%) from the survey on the Status of Nutrition and Health of the Chinese People in 2009–2010, which corresponded to 325 million hypertensive adults (above 18 years old) [3,31]. However, our findings were higher than the prevalence of hypertension, which were 15.7% (male: 17.4%; female: 13.5%), in Jiangxi province in 2013–2014 [32].

Our study showed that difference wasn’t statistically significant in age-standardized prevalence between females and males, which was not consistent with some previous studies, which showed that the hypertension prevalence in males was different with that of females [32,33,34,35]. Analyses of the participants revealed that female participants in this study were significantly older than males (59 vs. 53, *p* < 0.001), and they have a significantly higher prevalence rate of DM as compared to males. These and other related factors may contribute to the confounding bias.

Most current reports have indicated that smoking was a risk factor for hypertension. In this study, we also concluded that previous smoking could be a risk factor positively associated with the prevalence of hypertension as compared to never smoking, and such factor affects both genders significantly. Nevertheless, several other studies suggested that current smokers were more likely to suffer hypertension [34,35]. These different observations may largely be attributed to the use of different standards to classify participants for their previous and present smoking status. In addition, our study also revealed that previous smoking has more serious impact on females with about 10-fold increased risk of suffering hypertension than males. A recommendation from the eighth National Committee (JNC-8) was the modification of personal lifestyles including giving up smoking which might be necessary for all individuals to prevent hypertension [36].

Our study showed that DM was significantly associated with higher odds of hypertension for both genders. Some other studies indicated that insulin resistance was most likely the mechanism that links this cluster of disorders [37]. Insulin resistance, especially hepatic insulin resistance, has been determined to have a critical role in the development of atherosclerosis [38]. Contrary to the risk of ever smoking leading to hypertension, males with DM were likely to have more risk for hypertension (>2 times) than females. Thus, it is important to monitor blood pressure more frequently for male diabetics.

This study also revealed that overweight and obesity are higher risk factors for hypertension in both genders. Regardless gender difference, the higher BMI was directly linked with the higher prevalence of hypertension. This finding was consistent with several studies indicating overweight and obesity to be the important causes of hypertension [39,40,41]. Therefore, the health intervention program has important practical significance by aiming at maintaining body shape and fitness.

In this study, family history of CVD was a risk factor of hypertension only in males and there is no direct explanation presently known for this observation. Because of the importance of the association between family history of CVD and hypertension, more in-depth research with a large participant enrollment from different regions needs to be conducted to validate this finding and also to understand why males were more vulnerable to be affected by family CVD history than females. In addition, this study showed that the daily consumption of vegetables was a risk factor for hypertension only in females, while sleeping time and consumption of fruits were protective factors. Differences in age structure, economic status, and educational levels between male and female residents may be the cause of this phenomenon, which requires more research in the future to confirm the finding. Furthermore, although previous reports suggested high-salt diet to be a significant risk of increased hypertension prevalence, however, this was not observed in the present study [33,42,43]. One possible cause of this disputed observation may be due to recall bias and message bias resulting from self-reported diet.

## 5. Strength and Limitations

There are several strength of this study. To ensure the high quality of data collection and analysis, all the members of our survey team involved in the study have undergone a strict training procedure, and all survey procedures were completed in accordance with the standard protocol and measurement instructions. In addition, the questionnaire used in the study has been widely applied by a variety of research teams in China with good validity and reliability. Given that our analyses excluded subjects suffering from severe chronic diseases, fever in the last 15 days, and pregnant females, it is reasonable to believe the true hypertension prevalence might be underestimated in this Chinese adult population. Another strength of this study is that several more sorts of life behavior factors and their relationship to hypertension were employed for data analyses in this study to ensure a more accurate conclusion for hypertension prevalence and influencing factors. Finally, the data could be updated in real time to provide data basis for the development of health policy, further research, and explorations.

Note that this study also subjects to its limitations. A cross-sectional study precludes us from determining a cause–effect relationship between influencing factors and hypertension. Data collected in this study were limited to some communities in Nanchang city of Jiangxi Province so that these results may not be generalizable to other regions and locations. Furthermore, hypertension could be defined in different ways including self-reporting method and basing on patient diagnostic records or administrative databases, which limits the power to compare prevalence figures between different studies. Additionally, only BMI and WC (waist circumference)—traditional survey methods used when there is a lack of physiological and laboratory indicators—were measured in this study. Finally, recall bias and message bias might also exist in this study due to self-report lifestyle conditions.

## 6. Conclusions

The hypertension prevalence obtained in this survey in Nanchang, China was lower than that of national level. Several risk factors were found to be positively associated with hypertension in both males and females including previous smoking, DM, and overweight and obese BMIs. In particular, diabetic males and females who previously smoked need to be monitored more frequently in order to reduce their hypertension prevalence. These findings will form the baseline information for the development of more gender-specific intervention approaches to prevent and control adult hypertension more effectively in future. Also, findings from this study suggest the need to enhance health education and promotion for community residents, especially to encourage them to change their lifestyle behaviors in order to protect the high-risk population from hypertension.

## Figures and Tables

**Table 1:** Definitions of recoded independent variables.

Variables	Categories
Age group	18–39, 40–59, ≥60 years old
Location	Urban, Rural
Marital status	Single, Married or cohabitation, Widowed and divorced or separation
Educational level	Illiterate or primary school, High school, College
Occupation	Manual workers, Officials, Venders, Unemployed and other
Smoking history	Current smokers, Former smokers, Never-smokers
Alcohol consumption	Regular drinkers, Non-drinkers
Physical exercise (Thousand step equivalents of task)	No exercise
	Qualified physical exercise (≥6)
	Not qualified physical exercise (<6) [20]
DM	No (FPG < 7.0 mmol/L and 2h-PG < 11.1 mmol/L, a self-reported history of not DM)
	Yes (FPG ≥ 7.0 mmol/L and/or 2h-PG ≥ 11.1 mmol/L, a self-reported history of DM, or the use of diabetic medication) [21]
BMI (kg/m^2^)	Underweight (<18.5), Normal (18.5–24), Overweight (24–28), Obese (≥28) [19,22]
Abdominal obesity (cm)	No (waist circumference (<90 for male, <85 for female))
	Yes (waist circumference (≥90 for male, ≥85 for female)) [16]
Amount of sleep time per night (hour)	<7, ≥7 [23]

FPG: fasting plasma glucose; 2h-PG: two-hour postprandial plasma glucose; BMI: body mass index. DM: diabetes mellitus.

**Table 2:** Characteristics of the study sample stratified by sex.

Characteristics	Total (n = 2516) ^a,b^	Male (n = 1238) ^a,b^	Female (n = 1278) ^a,b^	*p*
Age (years) ^c^	56 (41–67)	53 (40–67)	59 (45–68)	*p* < 0.001
Age group				
18–39	518 (20.6)	305 (24.7)	213 (16.7)	*p* < 0.001
40–59	865 (34.4)	430 (34.7)	435 (34.0)	
≥60	1133 (45.0)	503 (40.6)	630 (49.3)	
Location				
Urban	1272 (50.6)	620 (50.1)	652 (51.0)	NS (0.64)
Rural	1244 (49.4)	618 (49.9)	626 (49.0)	
Marital status				
Married or cohabit	2157 (85.7)	1103 (89.1)	1054 (82.5)	*p* < 0.001
Unmarried	146 (5.8)	94 (7.6)	52 (4.1)	
Widowed, divorced, or separated	213 (8.5)	41 (3.3)	172 (13.4)	
Educational level				
Illiterate or primary school	1568 (62.3)	696 (56.2)	872 (68.2)	*p* < 0.001
High school	612 (24.3)	335 (27.1)	277 (21.7)	
University	336 (13.4)	207 (16.7)	129 (10.1)	
Occupation				
Manual workers	245 (9.7)	191 (15.4)	54 (4.2)	*p* < 0.001
Officials	290 (11.5)	176 (14.2)	114 (8.9)	
Venders	268 (10.7)	156 (12.6)	112 (8.8)	
Unemployed and other	1713 (68.1)	715 (57.8)	998 (78.1)	
Smoking history				
Current	647 (25.7)	595 (48.1)	52 (4.1)	*p* < 0.001
Past	100 (4.0)	90 (7.3)	10 (0.8)	
Never	1769 (70.3)	553 (44.6)	1216 (95.1)	
Alcohol consumption				
No	1986 (78.9)	813 (65.7)	1173 (91.8)	*p* < 0.001
Yes	530 (21.1)	425 (34.3)	105 (8.2)	
Physical exercise				
No	452 (18.0)	286 (23.1)	166 (14.0)	*p* < 0.001
Qualified	1139 (45.3)	478 (38.6)	661 (51.7)	
Not qualified	925 (36.7)	474 (38.3)	451 (35.3)	
DM				
No	2297 (91.3)	1152 (93.1)	1145 (89.6)	*p* < 0.005
Yes	219 (8.7)	86 (6.9)	133 (10.4)	
BMI				
Normal	1579 (62.8)	766 (61.9)	813 (63.6)	*p* < 0.01
Thin	145 (5.8)	62 (5.0)	83 (6.5)	
Overweight	642 (25.5)	348 (28.1)	294 (23.0)	
Obesity	150 (5.9)	62 (5.0)	88 (6.9)	
Abdominal obesity				
Yes	2014 (80.1)	943 (76.2)	1071 (83.8)	*p* < 0.001
No	502 (19.9)	295 (23.8)	251 (16.2)	
Family history of CVD				
Yes	2152 (85.5)	186 (15.0)	178 (14.0)	NS (0.44)
No	364 (14.5)	1052 (85.0)	1100 (86.0)	
Amount of sleeping (hour/night)				
<7	599 (23.8)	256 (20.7)	343 (26.8)	*p* < 0.001
≥7	1917 (76.2)	982 (79.3)	935 (73.2)	
Salt intake (g/day) ^c^	5.07 (3.93–7.38)	4.92 (3.93–7.27)	5.07 (3.93–7.38)	NS (0.96)
Fresh vegetables (50 g/day) ^c^	5 (5–8)	5 (5–8)	5 (5–9)	*p* < 0.05
Fresh fruits (50 g/day) ^c^	3 (2–5)	3 (2–5)	3 (2–5)	*p* < 0.005

NS: not statically significant (*p* > 0.05). ^a^ Value = n (%), indicate that number of cases and proportions stratified by categorical variables of males or females; ^b^ Continuous variables were tested for normality, and they were presented as mean ± standard deviation if they were normally distributed) or presented as median (lower quartile-upper quartile) if they were not normally distributed; ^c^ Continuous variables. DM: diabetes mellitus. BMI: body mass index. CVD: cardiovascular diseases.

**Table 3:** The age-standardized hypertension prevalence stratified by sex and age.

Age Group	Total (n = 2516)		Male ^a^ (n = 1238)		Female ^a^ (n = 1278)	
	N ^b^	Prevalence (95% CI)	N ^b^	Prevalence (95% CI)	N ^b^	Prevalence (95% CI)
18–39 ^c^	20	3.9 (2.2–5.5)	13	4.3 (2.5–6.0)	7	3.3 (1.8–4.8)
40–59 ^c^	163	18.8 (16.2–21.5)	76	17.7 (15.1–20.2)	87	20.0 (17.3–22.7)
≥60 ^c^	539	47.6 (44.7–50.5)	248	49.3 (46.4–52.2)	291	46.2 (43.3–49.1)
Sum ^c^	722	28.7 (26.9–30.5)	337	27.2 (25.5–29.0)	385	30.1 (28.3–31.9)
Standardized prevalence		19.8 (18.2–21.3)		19.5 (18.0–21.1)		20.0 (18.5–21.6)

^a^ there were statistically significant differences in prevalence among different age groups, *p* < 0.001; ^b^ number of hypertensive; ^c^ there was no statistically significant difference in the hypertension prevalence between males and females regardless of any age group, *p* > 0.05. CI: confidence interval.

**Table 4:** Crude OR and 95% CI for Hypertension from univariate logistic regression ^a^.

Characteristics	Male (n = 1238)			Female (n = 1278)		
	N of Participants (%) (n = 1238)	N of Hypertension (%) (n = 337)	Crude OR (95% CI)	N of Participants (%) (n = 1278)	N of Hypertension (%) (n = 385)	Crude OR (95% CI)
Age Group						
18–39	305 (24.6)	13 (4.3)	1	213 (16.7)	7 (3.3)	1
40–59	430 (34.7)	76 (17.7)	4.822 (2.62–8.86) ^b^	435 (34.0)	87 (20.0)	7.357（3.34–16.20) ^b^
≥60	503 (40.7)	248 (49.3)	21.845 (12.20–39.11) ^b^	630 (49.3)	291 (46.2)	25.262（11.70–54.53) ^b^
Smoking history						
Never	553 (44.7)	148 (23.5)	1	1216 (95.1)	362 (30.8)	1
Current	595 (48.0)	140 (54.4)	0.842 (0.645–1.100)	52 (4.1)	16 (70.0)	1.048 (0.575–1.914)
Ever	90 (7.3)	49 (26.8)	3.27 (2.074–5.158) ^b^	10 (0.8)	7 (29.8)	5.505 (1.416–21.406) ^b^
Alcohol consumption						
No	813 (65.7)	227 (27.9)	1	1173 (91.8)	355 (30.3)	1
Yes	425 (34.3)	110 (25.9)	0.901 (0.691–1.176)	105 (8.2)	30 (28.6)	0.922 (0.593–1.433)
Physical exercise						
No	286 (23.1)	45 (15.7)	1	166 (13.0)	42 (25.3)	1
Qualified	478 (38.6)	171 (35.8)	2.983 (2.062–4.315) ^b^	661 (51.7)	213 (32.2)	1.404 (0.954–2.065)
Not qualified	474 (38.3)	121 (25.5)	1.836 (1.256–2.683) ^b^	451 (35.3)	130 (28.8)	1.196 (0.798–1.793)
DM						
No	1152 (93.0)	256 (22.2)	1	1145 (89.6)	265 (23.1)	1
Yes	86 (7.0)	81 (94.2)	56.7 (22.737–141.396) ^b^	133 (10.4)	120 (90.2)	30.653 (17.017–55.217) ^b^
BMI						
Normal	784 (63.3)	173 (22.1)	1	842 (65.9)	210 (24.9)	1
Thin	44 (3.6)	10 (22.7)	1.039 (0.503–2.145)	54 (4.2)	19 (35.2)	1.634 (0.915–2.918)
Overweight	348 (28.1)	125 (35.9)	1.980 (1.501–2.610) ^b^	294 (23.0)	114 (38.8)	1.906 (1.438–2.526) ^b^
Obesity	62 (5.0)	29 (46.8)	3.104 (1.833–5.255) ^b^	88 (6.9)	42 (47.7)	2.748 (1.758–4.294) ^b^
Abdominal obesity						
No	943 (76.2)	212 (22.5)	1	1071 (83.8)	281 (26.2)	1
Yes	295 (23.8)	125 (42.4)	2.535 (1.922–3.344) ^b^	207 (16.2)	104 (50.2)	2.839 (2.093–3.849) ^b^
Family history of CVD						
No	1052 (85.0)	264 (25.1)	1	1100 (86.1)	317 (28.8)	1
Yes	186 (15.0)	73 (39.3)	1.928 (1.392–2.670) ^b^	178 (13.9)	68 (38.2)	1.527 (1.099–2.122) ^b^
Amount of sleep (hour/night)						
<7	256 (20.7)	95 (37.1)	1	343 (26.8)	144 (42.0)	1
≥7	982 (79.3)	242 (24.6)	0.554 (0.414–0.742) ^b^	935 (73.2)	241 (25.8)	0.480 (0.370–0.622) ^b^
Salt intake (g/day) ^c^	1238 (100)	337 (27.22)	1.028 (0.992–1.066)	1278 (100)	385 (3013)	1.023 (0.990–1.057)
Fresh vegetables (50 g/day) ^c^	1238 (100)	337 (27.22)	1.105 (1.050–1.163) ^b^	1278 (100)	385 (3013)	1.099 (1.051–1.150) ^b^
Fresh fruits (50 g/day) ^c^	1238 (100)	337 (27.22)	0.911 (0.861–0.963) ^b^	1278 (100)	385 (3013)	0.877 (0.831–0.926) ^b^

^a^ Applied method of enter for univariate logistic regression; ^b^ Value of OR was significant from univariate logistic regression; ^c^ Continuous variables. OR: odds ratio. CI: confidence interval. DM: diabetes mellitus. BMI: body mass index. CVD: cardiovascular diseases.

**Table 5:** Adjusted OR and 95% CI for hypertension from multivariate logistic regression ^a^.

Characteristics	Male (n = 1238)		Female (n = 1278)	
	Model 1 ^b^	Model 2 ^c^	Model 1 ^b^	Model 2 ^c^
	Adjusted OR (95% CI)	Adjusted OR (95% CI)	Adjusted OR (95% CI)	Adjusted OR (95% CI)
Age Group				
18–39	1	1	1	1
40–59	4.102 (2.119–7.940) ^d^	4.717 (2.213–10.054) ^d^	5.599 (2.421–12.950) ^d^	5.69 (2.197–14.740) ^d^
≥60	17.319 (9.060–33.107) ^d^	20.591 (9.069–46.753) ^d^	15.357 (6.743–34.975) ^d^	13.391 (5.072–35.353) ^d^
Smoking history				
Never	1	1	1	1
Current	1.05 (0.743–1.485)	1.033 (0.728–1.467)	0.928 (0.455–1.891)	0.969 (0.473–1.988)
Ever	1.887 (1.077–3.306) ^d^	1.921 (1.090–3.384) ^d^	14.78 (2.307–94.699) ^d^	11.425 (1.873–69.682) ^d^
Alcohol consumption				
No	1	1	1	1
Yes	0.904 (0.644–1.270)	0.891 (0.632–1.256)	0.734 (0.429–1.256)	0.752（0.438–1.293)
Physical exercise				
No	1	1	1	1
Qualified	1.546 (0.967–2.471)	1.53 (0.950–2.466)	0.883 (0.551–1.413)	0.861 (0.535–1.387)
Not qualified	1.25 (0.784–1.994)	1.258 (0.779–2.031)	0.937 (0.574–1.527)	0.933 (0.571–1.527)
DM				
No	1	1	1	1
Yes	41.235 (15.706–108.258) ^d^	41.192 (15.720–107.936) ^d^	21.027 (11.388–38.823) ^d^	20.202 (10.896–37.456) ^d^
BMI				
Normal	1	1	1	1
Thin	0.909 (0.392–2.105)	0.894 (0.386–2.072)	1.33 (0.656–2.695)	1.346 (0.660–2.748)
Overweight	1.917 (1.327–2.770) ^d^	1.89 (1.304–2.739) ^d^	1.666 (1.182–2.349) ^d^	1.641 (1.161–2.320) ^d^
Obesity	3.362 (1.630–6.934) ^d^	3.292 (1.575–6.881) ^d^	2.159 (1.207–3.862) ^d^	2.136 (1.189–3.839) ^d^
Abdominal obesity				
No	1	1	1	1
Yes	1.763 (1.189–2.614) ^d^	1.827 (1.221–2.734) ^d^	1.346 (0.895–2.025)	1.332 (0.881–2.014)
Family history of CVD				
No	1	1	1	1
Yes	1.592 (1.027–2.469) ^d^	1.579 (1.014–2.458) ^d^	1.263 (0.831–1.919)	1.252 (0.821–1.911) ^d^
Amount of sleeping (hour/night)				
<7	1			
≥7	0.706 (0.492–1.014)	0.723 (0.501–1.042)	0.699（0.511–0.956) ^d^	0.702 (0.510–0.965) ^d^
Salt intake (g/day) ^e^	0.975 (0.931–1.021)	0.972 (0.927–1.019)	1.002（0.963–1.043)	1.004 (0.964–1.046)
Fresh vegetables (50 g/day) ^e^	1.016 (0.952–1.084)	1.016 (0.951–1.084)	1.057（1.002–1.114) ^d^	1.055 (1.000–1.113) ^d^
Fresh fruits (50 g/day) ^e^	0.948 (0.883–1.018)	0.947 (0.882–1.016)	0.93（0.872–0.993) ^d^	0.935（0.875–0.998) ^d^

^a^ All applied method of enter for multivariate logistic regression; ^b^ adjusted by all the risk factors listed at this table; ^c^ model1 + age group, location, marital status, educational level, occupation; ^d^ Values of OR are significant; ^e^ Continuous variable. OR: odds ratio. CI: confidence interval. DM: diabetes mellitus. BMI: body mass index. CVD: cardiovascular diseases.
